# Analysis of the Endogenous Peptidomes of Different Infant Formula Types and Human Milk

**DOI:** 10.3390/foods10112579

**Published:** 2021-10-26

**Authors:** Michele Wölk, Corinna Gebauer, Ralf Hoffmann, Sanja Milkovska-Stamenova

**Affiliations:** 1 Institute of Bioanalytical Chemistry, Faculty of Chemistry and Mineralogy, Universität Leipzig, Deutscher Platz 5, 04103 Leipzig, Germany; michele.woelk@uni-leipzig.de; 2 Center for Biotechnology and Biomedicine, Universität Leipzig, Deutscher Platz 5, 04103 Leipzig, Germany; 3 Department of Paediatrics, Division of Neonatology, University of Leipzig Medical Center, Liebigstr. 20 a, 04103 Leipzig, Germany; Corinna.Gebauer@medizin.uni-leipzig.de

**Keywords:** β-casein, breast milk, caprine milk, goat formula, infant feeding, liquid formula, native peptides

## Abstract

Infant formula (IF) is a commonly used replacement whenever mother’s own milk is not available. Most IFs are based on cow milk (powders, liquids). Alternatives, based on other sources such as goat milk or plants, exist. Independent of the source, IF production and composition are strictly regulated. Besides proteins, minerals, and lipids, milk contains a variety of endogenous peptides. Whereas the human milk peptidome has been studied intensively, the peptidomes of IFs have been mostly neglected. This study investigated the peptidomes of different types of first stage IF, including cow milk-based powders and liquids, and powdered goat milk-based IF, highlighting major similarities and differences to human milk. Extracted native peptidomes were analyzed by nanoRPC-ESI-MS/MS using two different fragmentation techniques allowing the confident identification of 1587 peptides. β-Casein peptides dominated in all samples. Interestingly, powdered and liquid cow milk-based IFs differed in the numbers of β- and α_S1_-casein peptides, indicating processing-derived variations. However, the peptidomes of cow and goat milk-based IF appeared to be more comparable to each other than to human milk. Despite an overlap in the major source proteins, many peptide sequences were different, i.e., species-specific. Remarkably, the data indicate that the human milk peptidome might be donor-specific as well.

## 1. Introduction

Mother’s own milk (MOM) is the best choice for infant feeding as it suits all nutritional requirements of newborns [1]. The World Health Organization (WHO) recommends exclusive breastfeeding in the first six months after birth and continued breastfeeding up to the age of at least two [2]. However, more than half of breastfed infants receive MOM exclusively only for three months, while 20% of all babies from high income countries are not breastfed [3]. Instead, many infants are fed with so-called infant formula (IF), which is produced mostly from cow milk and adjusted to the composition of human milk following strict guidelines [4,5]. As the nutritional requirements of infants vary, diverse forms of IFs are available on the market, mostly as powders. Among these also are products with specialized composition, such as hydrolyzed or anti-reflux IF, and products based on plants (soy) or goat milk [6,7]. Besides powders, IFs are also sold as liquid concentrates or ready-to-drink products [4]. Independent of the source material, the protein and fat contents of IF have to be adjusted to human milk. For example, the casein to whey ratio differs between human (40:60), cow (80:20), and goat milk (70:30) [1]. Thus, cow and goat milk-based IFs are supplemented with α-lactalbumin (the major whey protein in human milk), whereas the overall protein amount has to be reduced to the lower level of human milk [8,9].

Generally, IF production involves several heat treatments to ensure the microbiological safety of the final product [4]. Ready-to-drink IFs are produced by fewer processing steps and lower heat treatments than powders [10]. Thermal processing might affect the composition of the peptidome, for example by triggering Maillard reactions [11]. At the protein level, these reactions block lysine residues and thus decrease the digestibility of the modified proteins [12]. However, thermal processing also affects the endogenous peptides present in cow milk [13]. Milder processing conditions might be more beneficial for infants.

Due to their important bioactivities, such as anti-hypertensive, anti-inflammatory, anti-diabetic, antioxidant properties, and opioid activities, milk-derived peptides are of general interest [14]. While endogenous peptides present in human milk and their biological functions have been studied [15,16,17], less attention has been paid to endogenous peptides present in IFs. As production regulations for IFs do not consider the peptidome, they will significantly differ from human milk. Only two studies have compared the peptidomes of human milk and IFs [18,19]. We hypothesized that the differences in the milk sources used for IF production and the different processing conditions applied to powders and ready-to-drink IFs affect the peptidomes of the final products and therefore the aim of this study was to qualitatively characterize the peptidomes of different types of IF and to identify similarities and differences compared to the peptidome of human milk. To the best of our knowledge, studies comparing the native peptidomes of ready-to-drink IFs, powdered IFs derived from cow or goat milk, and human milk are missing.

Accordingly, here the peptidomes of two powdered and two liquid (ready-to-drink) cow milk-based IFs, two powdered goat milk-based IFs, and two human milk samples were analyzed in parallel using nanoRPC-ESI-MS/MS. This study considered only peptides that were independently identified by two different fragmentation techniques, i.e., collision induced (CID) and electron transfer dissociation (ETD), with confident scores. As expected, the human milk peptidome was dominated by β-casein-derived peptides. Interestingly, donor-specific peptide patterns were observed. Furthermore, the peptidomes of powdered and ready-to-drink IF differed in the numbers of peptides originating from β- and α_S1_-casein, i.e., powders contained more α_S1_-casein-derived peptides and liquids more β-casein-derived peptides. The goat milk-based IF contained similar numbers of peptides originating from β- and α_S2_-casein. Although β-casein peptides dominated in cow, goat, and human milk, the peptidomes of cow and goat milk based IFs differed significantly from human milk.

## 2. Materials and Methods

### 2.1. Milk Samples

#### 2.1.1. Infant Formula Samples

Two types of cow milk-based first stage IF from two different brands (1 and 2) were purchased at local supermarkets as powders (IF P1 and IF P2) and liquid ready-to-drink IFs (IF L1 and IF L2). Additionally, two brands of goat milk-based first stage powders (IF G1 and IF G2) were obtained (Table S1). It should be noted that only these two brands of goat milk-based first stage powders and the two brand pairs of cow milk-based powdered and liquid infant formulas were available on the German market, thereby limiting the number of samples included in this study. Powdered IFs were prepared with purified water in accordance with the manufacturers’ information provided on the packages. Ready-to-drink IFs were heated in a water bath up to 37 °C as written on the package. All samples were cooled to room temperature before storage at −80 °C until further analysis.

#### 2.1.2. Human Milk Samples

Human milk was donated from two volunteers who delivered term matched for age, lactation month, and infant gender (Table S2). Importantly, milk samples were collected before breastfeeding in the morning (between 6:30 and 8 am), and temporarily stored at −20 °C until provided to the milk bank, which stored them at −80 °C until analysis. 

### 2.2. Peptide Extraction

Samples were thawed on ice and the native peptidomes were extracted from all samples (six different first stage infant formulas and two donor milk samples) in triplicates as described previously [13]. Briefly, methanol (375 µL) and chloroform (750 µL) were added to 50 µL of a sample and incubated (1 h, 4 °C). After addition of water (625 µL), the sample was incubated (10 min, 4 °C), centrifuged (10 min, 10,000× *g*, 4 °C), the organic phase removed, and centrifuged again using the same conditions. The peptide-containing aqueous phase was dried under vacuum. Peptides were reconstituted in 100 µL aqueous acetonitrile (3%, *v*/*v*) containing 0.1% (*v*/*v*) formic acid, desalted by solid-phase extraction (SPE, Oasis HLB, 1cc, 30 mg, Waters GmbH, Eschborn, Germany), and the eluate dried under vacuum. Peptides were reconstituted in aqueous acetonitrile (3%, *v*/*v*) containing formic acid (0.1%, *v*/*v*). Peptide concentrations were estimated on a NanoPhotometer NP80 (IMPLEN, Munich, Germany, λ = 280 nm). All chemicals used in this study are listed in Table S3.

### 2.3. Nano Liquid Chromatography Tandem Mass Spectrometry

#### 2.3.1. UPLC-ESI-LTQ-Orbitrap

Samples were analyzed on a nanoAcquity UPLC (Waters GmbH) coupled on-line to an LTQ Orbitrap XL ETD mass spectrometer equipped with a nano ESI-source (Thermo Fisher Scientific, Bremen, Germany) using previously reported LC and MS conditions [13]. Briefly, LTQ Orbitrap XL ETD settings were: capillary temperature 200 °C, ion spray voltage 1.4 kV (Pico-Tip^TM^ on-line nano-ESI emitter, New Objective, Berlin, Germany), *m/z* range 400 to 2000 (resolution of 60,000 at *m/z* 400). Tandem mass spectra were recorded in the linear ion trap using data-dependent acquisition (DDA) for the six most intense signals (DDA top 6) in electron transfer dissociation (ETD) mode with an isolation width of 2 *m/z* units, activation time 100 ms, default charge state 2, intensity threshold of 500 counts, and a dynamic exclusion window of 60 s.

#### 2.3.2. UPLC-ESI-QTOF

Peptides were separated on a nanoAcquity UPLC and analyzed on-line on a Synapt G2-S*i* mass spectrometer equipped with a nano-ESI source (Waters GmbH). Eluents A and B were water and acetonitrile, respectively, containing 0.1% (*v*/*v*) formic acid. Peptides were trapped (nanoAcquity Symmetry C18-column, internal diameter 180 µm, particle diameter 5 µm, length 2 cm) at a flow rate of 5 µL/min (1% eluent B), and separated on a BEH 130 column (inner diameter 75 µm, particle diameter 1.7 µm, length 10 cm, 30 °C) using a flow rate of 0.3 µL/min. Separation was achieved by linear gradients from 1% to 40% eluent B within 89 min and to 85% eluent B within 5 min. The mass spectrometer was operated in positive ion mode and tandem mass spectra were acquired in high definition data directed analysis mode (HD-DDA) using T-Wave™ ion mobility with wideband enhancement [20] using the following instrument settings: capillary voltage 3 kV, sample cone 30 V, source offset 80 V, temperature 100 °C, cone gas flow 20 L/h, and nanoflow gas 0.2 bar. A GluFib (*m/z* 785.84204) solution was sprayed as lock mass reference at a flow of 0.5 µL/min and acquired in intervals of 30 s with a scan time of 0.2 s. Full scan spectra were recorded in a *m/z* range from 300 to 2000 with a scan time of 0.2 s and a threshold of 1000 counts. Fragmentation was induced by a collision energy ramp from 31.7 to 44.2 V for *m/z* 1150. Tandem mass spectra were acquired from *m/z* 50 to 5000 with a scan time of 0.4 s for the 5 most intense precursor ions, using a dynamic exclusion of 6 s for a window of ±250 mDa. TWIMS settings were as reported earlier [21] and a wideband enhancement was applied to increase the sensitivity for singly charged fragment ions.

### 2.4. Data Procesing

Acquired data from both mass spectrometers were processed with PEAKS Studio 10.5 (Bioinformatics Solutions Inc., Waterloo, ON, Canada) using instrument-dependent parameters: precursor mass tolerance 10 ppm (30 ppm); fragment mass tolerance 0.8 Da (0.1 Da), and fragmentation ETD (CID) for LTQ Orbitrap XL ETD (Synapt G2-S*i*). Database settings included no enzyme, dynamic modifications including oxidation of methionine (+15.99 Da) and phosphorylation of serine, threonine, and tyrosine (+79.96 Da, Phospho), and a false discovery rate of 1%. Human-, bovine-, and goat-specific databases were downloaded from UniProt as FASTA files considering only reviewed proteins for the database searches: Bos taurus (October 2020, 6013 proteins), Capra hircus (October 2020, 120 proteins) and Homo sapiens (October 2020, 20,385 proteins). The confident identification of native peptides considered only peptides proposed by both CID and ETD in two different replicates for at least one brand/donor.

## 3. Results

### 3.1. Identification of Native Peptides and Their Source Proteins

**Figure 1:**
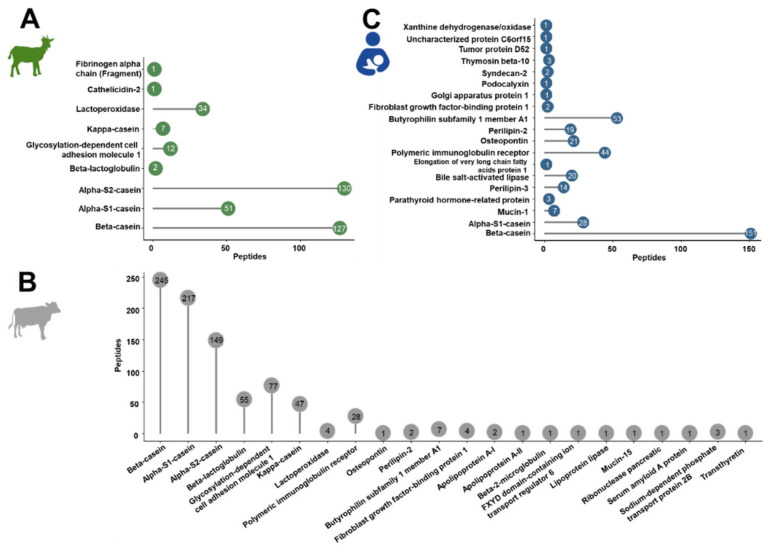
Overall number of peptides identified for each source protein in goat (**A**) and cow milk-based IF (**B**) and in human milk (**C**).

The confident profiling of the peptides extracted from powdered and liquid IFs or human milk relied on CID and ETD using different mass spectrometers considering only peptides proposed by both analyses. Relying in the database search only on bovine, goat and human proteins marked as reviewed in the Uniprot database, a total of 1587 peptides were identified. Most peptides were identified in cow milk-based IF (849 peptides from 22 bovine proteins) compared to 365 peptides corresponding to nine proteins in goat milk-based IF and 373 peptides from 19 proteins in human milk (Tables S4–S6, Figure 1).

#### 3.1.1. Goat Milk-Based IF

**Figure 2:**
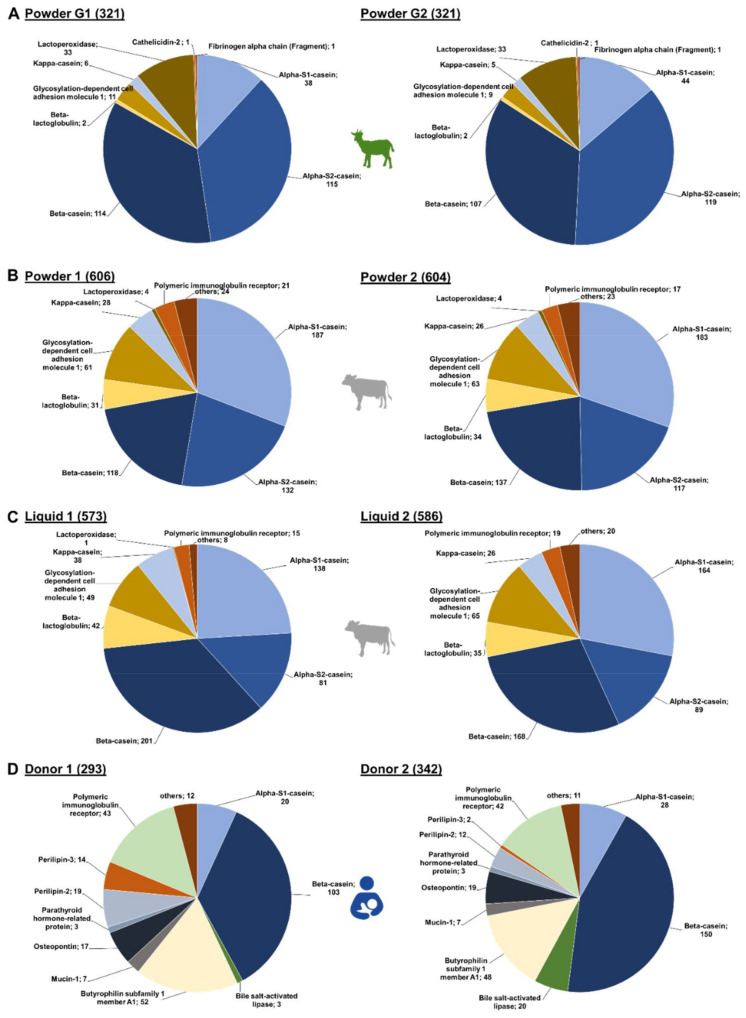
Number of peptides identified for each source protein in the individual brands of (**A**) goat (Powder G1 and G2), (**B**) cow milk-based powdered IF (Powder 1, Powder 2) and (**C**) cow milk-based liquid IF ( Liquid 1, Liquid 2) as well as in (**D**) human milk (Donor 1 and Donor 2).

In the IF samples based on goat milk 130 peptides originated from α_S2_-casein and 127 peptides from β-casein, while α_S1_-casein and lactoperoxidase were represented by 51 and 34 peptides, respectively (Figure 1A). Furthermore, a few peptides corresponded to low-abundant proteins, i.e., glycosylation-dependent cell adhesion molecule 1 (12 peptides), κ-casein (7), β-lactoglobulin (2), cathelicidin-2 (1), and fibrinogen α-chain (fragment) (1). Both goat brands showed very similar peptide profiles (Figure 2A,) with ~76% of all peptides identified in both samples and similar numbers of peptides derived from α_S2_- and β-casein representing almost 70% of the accessible peptidome of each brand. These two proteins accounted together with α_S1_-casein and lactoperoxidase for 93.4% and 94.4% of all identified peptides in brands G1 and G2, respectively. Importantly, only two peptides from β-lactoglobulin were identified in goat milk-based IFs.

#### 3.1.2. Cow Milk-Based IF

The detected endogenous peptidome was dominated by 245 peptides originating from β-casein, 217 peptides corresponding to α_S1_-casein, and 149 α_S2_-casein-derived peptides, while only 47 and 55 peptides originating from κ-casein and β-lactoglobulin, respectively, were detected (Figure 1B). Among the milk fat globular membrane proteins, most peptides corresponded to glycosylation-dependent cell adhesion molecule 1 (77) and polymeric immunoglobulin receptor (28).

In powdered IFs, slightly more peptides were identified than in the liquid IFs (Figure 2B,C). The overlap of identified peptides was higher between the powder IFs (75.9%) than in the liquid IFs (61.9%). Interestingly, the overlap in peptides identified in powder and liquid IFs from the same brand was also low (52.3% brand 1 and 62.1% brand 2). Notably, liquid IFs contained more β-casein-derived peptides, but less α_S1_- and α_S2_-casein peptides in comparison to powdered IF. Consequently, the difference between the peptidomes of powdered and liquid IFs are mainly based on variations among these three caseins, which represent up to 73.3% of all identified peptides. Interestingly, similar numbers of peptides originating from β-lactoglobulin (31 to 35 peptides), κ-casein (26 to 28 peptides), and glycosylation-dependent cell adhesion molecule 1 (61 to 65 peptides) were identified in both powdered IFs and liquid IF from brand 2 (Figure 2B,C). The liquid IF from brand 1 contained more peptides from β-lactoglobulin (42) and κ-casein (38), but less from glycosylation-dependent cell adhesion molecule 1 (49) (Figure 2C left).

#### 3.1.3. Human Milk

A total of 373 endogenous peptides from 19 proteins were identified (Figure 1C) with almost half of them corresponding to β-casein (151). Additionally, many peptides derived from butyrophilin subfamily 1 member A1 (53) and polymeric immunoglobulin receptor (44). Further peptides originated from α_S1_-casein (28), osteopontin (21), bile salt-activated lipase (20), perilipin-2 (19), perilipin-3 (14) and a few other proteins represented by less than ten peptides.

Despite a 70.2%-similarity of both human milk peptidomes, a few donor-specific differences appeared to be interesting (Figure 2D). About 15% more peptides were identified in milk obtained from donor 2 (342) compared to donor 1 (293), which was mostly attributed to the number of β-casein-derived peptides. However, variations were also observed for other proteins. While only three peptides corresponding to bile salt-activated lipase were found in the sample from donor 1, 20 peptides originating from this protein were identified in the sample from donor 2. In contrast, the milk from donor 1 contained 33 peptides derived from perilipin-2 and perilipin-3 compared to 14 detected in the sample of donor 2. Peptide numbers corresponding to the other source proteins were similar for both donors.

### 3.2. Comparison of the Peptidomes

#### 3.2.1. Peptides

**Figure 3:**
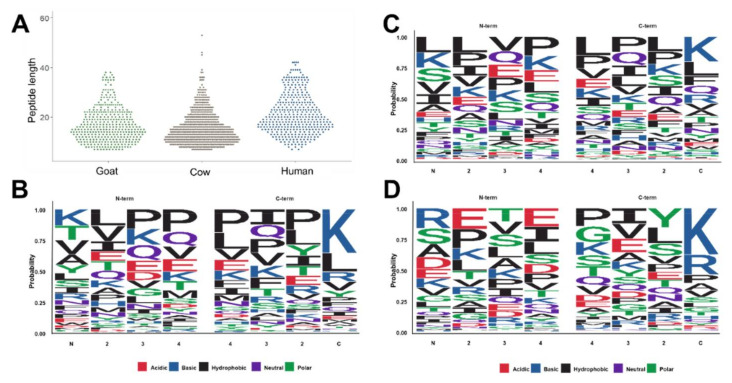
Peptide length distribution for all peptides identified in goat and cow milk-based IF and inhuman milk (**A**). Cleavage patterns for the four N-terminal (residues 1 to 4) and the four C-terminal residues observed in goat (**B**) and cow milk-based IF (**C**) and in human milk (**D**). Cleavage patterns were generated by using ggseqlogo within R [22].

The peptides identified in this study reflect the native peptidomes present in the samples prior to digestion within an infant’s stomach. In all three sample types, the shortest peptides identified by our analytical strategy were seven residues long, while the longest peptides identified in cow, goat, and human samples were 53, 38, and 42 residues long (Figure 3A). The average peptide lengths were significantly longer in human milk with 21.2 residues than in cow (15.8) and goat milk-based IF (17.1). In order to evaluate the composition of endogenous peptides, the four N-terminal and the four C-terminal residues of alle peptide sequences were plotted using the sequence logo package within R [22] (Figure 3B–D). In goat milk-based IF, the N-terminal sequence was dominated by Lys, Thr, Val, Ala, and Tyr counting together for a probability of 0.5 with the N-terminus often followed by a hydrophobic amino acid, such as Leu, Val or Ile (Figure 3B left). Lys appeared to be the main amino acid at the C-terminus followed by Leu and Arg (Figure 3B right). In cow milk-based IF, Leu, Lys, Ser, Val and Ile were more common at the N-terminus, while Lys and Leu were most frequent at the C-terminus, followed by Phe, Gln and Arg (Figure 3C). In human milk Arg, Ser, Ala and Asp were enriched at the N-terminus, whereas the C-terminal residues were typically Lys and Arg (Figure 3D).

Among the peptides identified in this study, 36 were common between the cow and goat milk peptidomes, i.e., four peptides derived from α_S1_-casein, 13 peptides from α_S2_-casein peptides, 15 peptides from β-casein, and four peptides from lactoperoxidase. Only peptide LPIIQKLEPQIA of perilipin-2 was shared between the peptidomes of cow and human milk. No peptides overlapped among all three species.

#### 3.2.2. Common Source Proteins

The peptidomes of all three species were dominated by peptides originating from β-casein and to a lower content from α_S1_-casein (Figure 1 and Figure 2). The sequence coverage of β-casein ranged from ~92% in cow to ~85% in goat and human samples (Figures S1–S3). As goat and bovine β-caseins have a rather high sequence homology of ~91%, it is not surprising that the detected peptides corresponded to similar regions with most peptides originating from the C-terminal sequence of β-casein (“hot spot”) starting around residues Val185 in the goat and Val177 in the cow sequence (Figures S1 and S2). The other peptides originated mostly from the N-terminal region of β-casein until Ala70 in goat and Ser72 in cow. Moreover, several goat milk derived peptides were detected in the region from Leu92 to Pro153. Importantly, the two brands of goat milk based IF were very similar and differed only in the sequence corresponding to Tyr129 to Thr169 of β-casein. In goat, the C-terminus of β-casein-derived peptides contained mostly Lys, Val, and Leu/Ile, whereas Val, Leu, and Arg were most frequent at the N-terminus (Figure S4A). Bovine β-casein was mostly covered by peptides, and only peptides corresponding to Leu73 to Gln87 were not detected (Figure S2). Both powdered samples showed similar sequence coverages by similar peptide numbers, while regions Ile45 to Ala68 and Val177 to Leu206 were covered by more peptides (Figure S2). Interestingly, the region Val170 to Gln175 was missed in sample L2. The N-terminus of all β-casein-derived peptides detected in the four studied cow milk-based IF carried mainly Ser, Leu, Val, and Lys, whereas Gln, Lys, Pro, and Phe were enriched at the C-terminus (Figure S4B).

In contrast to goat and cow, human milk β-casein-derived peptides originated mainly from three N-terminal (Arg16 to Pro57), central (Ala88 to Lys132), and C-terminal regions (Tyr211 to Val226) with peptide numbers decreasing in this order (Figure S3). Interestingly, no peptides covered region Leu145 to Lys175. At the N-terminal side of peptides Arg, Val, or Glu were frequent, while Lys was prevalent at the C-terminus (Figure S4C).

The average lengths of β-casein-derived peptides slightly decreased from human (20.3) to goat (19) and further in cow milk samples (16.5) (Figure S5A). Similarly, the average length of α_S1_-casein peptides decreased much from human (24.1) to goat (15.5) and cow milk samples (15.1) (Figure S5B). In contrast, the sequence coverage of α_S1_-casein excluding the signal peptide decreased greatly from cow milk-based IF (75.4%) to goat milk-based IF (54.7%) and even further for human milk (42.2%). Besides these two caseins, no further proteins were common among the three species. However, cow and human samples shared further proteins including polymeric immunoglobulin receptor, osteopontin, perilipin-2, butyrophilin subfamily 1 member A1, and fibroblast growth factor-binding protein 1 (Figure 1).

## 4. Discussion

### 4.1. Identification of Endogenous Peptides

Breast milk is the gold standard in infant nutrition as it provides all nutrients required for the healthy development of a child leading to the WHO-recommendation for exclusive breastfeeding for the first six months after birth [2]. While many infants are not breastfed at all, especially in high income countries, about half of the other infants are exclusively breastfed only for the first three months [3]. While the composition of human milk varies over the lactation period [17], IFs can offer only average compositions close to human milk, for example pre-term, first stage (0 to 6 months), and follow up IFs. Most commercial products rely on cow milk [5] and are marketed mainly as powders to be dissolved in boiled water prior to feeding. However, some liquid products, such as ready-to-drink IFs, are also offered. There are also anti-reflux or hypoallergenic IF types with modified compositions for infants with reflux or protein intolerance/allergy. Furthermore, for infants intolerant or allergic to cow milk there are alternative formulations based on milk from other species, such as goat milk [7], or plants, such as soy [6]. Independent of the type and the source material, the composition of IFs has to match human milk and the requirements of the infant. Thus, protein contents are adjusted, individual proteins or fatty acids are added, and different processing conditions are applied. Although many studies have analyzed the composition of IF to human milk, little attention has been paid to endogenous peptides either present in the protein source or formed during processing.

Human breast milk contains a variety of endogenous peptides, at least partially preformed in the mammary glands, with important biological functions [15,16,17,19,23]. The endogenous peptidomes of cow milk [24] and cow milk-based powdered IF have also been reported [13]. As IF should reflect human milk composition, it is important to determine how its native peptidome differs among species and to reveal alterations induced during processing. This study aimed to investigate the peptidomes of first stage IFs (0 to 6 months) in powders and liquids based on cow milk and powdered from goat milk as well as to identify similarities and differences compared to the peptidome of human milk collected from two donors at the third lactation month. To the best of our knowledge, this is the first study exploring the peptidomes of different first stage goat and bovine milk-based IF types relative to the human milk peptidome.

Compared to our recent peptidomics report including a powdered first stage cow milk-based IF, the total number of 849 peptides identified in cow milk-based IF here was slightly higher, but the numbers of peptides identified in each sample were slightly lower [13]. Interestingly, more peptides originating from major milk proteins, i.e., caseins and β-lactoglobulin, were identified in the current study, while less peptides were identified for several minor milk proteins e.g., butyrophilin subfamily 1 member A1 and osteopontin [13]. However, the analyzed brands differed from the previous study and consequently the observed differences in the peptidomes might reflect altered production conditions. Nevertheless, this study, relying on two different fragmentation techniques, leads to a more confident identification of native peptides. Interestingly, the processing of cow milk-based IFs appears to alter the peptide profiles with α_S1_-casein peptides dominating powdered formula and β-casein liquid IFs, with the observations for powdered IF matching well to previous studies [13,19]. To the best of our knowledge, the peptidome of liquid IF has not been studied and compared to its corresponding powdered product from the same brand. Thus, the data may indicate processing-related differences in the peptidomes with liquid IF being closer to human milk in terms of β-casein-derived peptides. As powder and liquid pairs were obtained from the same manufacturer, we assume that the ingredients for both product types are very similar. Thus, it remains open whether the β-casein peptides degraded during processing of powdered IF or were generated due to remaining protease activity in the liquids. Importantly, the sequence coverage of β-casein was almost equal for powder and liquid IFs.

The number of peptides identified in goat milk-based IFs were rather low compared to bovine milk-based products due to the small size of the database containing only reviewed proteins. By using a larger database or considering the bovine database, the number of peptides should increase. However, we aimed for confident identifications and not for high numbers of identified peptides. Thus, the peptidome of goat milk-based IFs still offers a great potential for the discovery of further endogenous peptides.

This stringent criteria for identifying a peptide resulted also in much lower numbers than recently reported for human milk [15,17,19]. For example, Lopez et al. identified 3131 unique peptides in human milk collected from three donors (8 to 25 weeks postpartum) using centrifugal concentrators followed by LC-MS with DDA top 15 [19]. Despite the different analytical strategy and considering only peptides proposed by both CID and ETD in our approach, the different peptide numbers may also be explained by the different lactation stages and sample collections. Indeed, only 637 of the 3131 peptides were identical among all three mothers indicating a donor- or lactation stage-specificity [19]. Furthermore, the composition of human milk changes not only with the lactation stage (8–25 weeks vs. 12 weeks analyzed here), but also during the day and between fore or hind milk, which was not specified in the previous study. Remarkably, our data indicate that the human milk peptidome might be donor-specific, although this has to be taken with caution as milk samples from only two donors were analyzed. In particular, perilipin-3 and bile salt-activated lipase, which are both related to milk lipids, appeared to be interesting due to the significantly different levels in donors. Perilipin-3 is positively correlated with the total lipid content in human milk [25] while bile salt-activated lipase aids in lipid digestion [26]. Notably, Zhu et al. reported donor-specific patterns in the proteins and peptide profiles of milk samples from two donors analyzed over 16 weeks postpartum [17]. However, precise conclusions will require further studies focusing on milk from more donors for different lactation periods considering also the fat content and profiling the lipidome. Ideally, future studies should rely on a higher number of infant formulas as well. Importantly, our study did not aim for an in-depth characterization of the human milk peptidome, but to investigate major similarities and differences compared to the different types of IFs as the first step towards a better understanding of the importance of the peptidomes for the healthy growth and development of the infants.

### 4.2. Similarities and Differences between the Peptidomes

The peptidomes of all three species were dominated by β-casein-derived peptides and, at lower numbers, by peptides originating from α_S1_-casein. This corresponds well to previous studies on human milk [17,19], while we identified α_S1_-casein as the main peptide source in unprocessed cow milk [13]. Although goat milk-based IFs are gaining popularity, little is known about their peptidome. Only two powdered first stage IFs based on goat milk were available with both peptidomes being dominated by β- and α_S2_-casein-derived peptides. Goat milk-based IFs have mostly been studied in the context of their digestion behavior. For instance, the kinetic of protein digestion of human milk in a model system was closer to goat milk-based IF than to cow milk-based products [27]. Under in vitro digestion conditions caseins in goat milk-based IFs were better digested than those in cow milk-based IFs [28].

The general dominance of β-casein peptides in all three species relates to the abundance of this protein, as β-casein is the most abundant protein in human and goat milk and present at equal concentrations as α_S1_-casein in cow milk [1]. Interestingly, mapping the peptides derived from β-casein indicated that peptides in human milk originated from other regions than in IF samples. For goat and cow milk-based IFs this was mostly the C-terminal region, whereas in human milk most peptides derived from the N-terminus leading to a major difference in the peptidomes. Peptides derived from the C-terminal part of β-casein were also observed in simulated gastric digests of both types of IF [29]. In all three peptidomes α_S1_-casein-derived peptides were observed. Additionally, the IFs showed peptides originating from α_S2_-casein. Due to the absence of this protein in human milk, such peptides could not be identified in human milk [30], which represents a significant difference between IFs and human milk.

The dominance of casein-derived peptides can be explained by the activity of plasmin in human and cow milk [31]. Plasmin cleaves proteins C-terminal to Lys- and Arg-residues, which corresponds well to the high number of peptides with a C-terminal Lys or Arg residue. This pattern and the enrichment of certain N-terminal amino acids fit well to an earlier study on the formation of endogenous peptides within the human mammary glands [15]. Similarly, Lys and Leu were most frequently observed at the C-terminus of peptides identified in cow and goat milk-based IF. Although all three species shared many peptides carrying a Lys residue at the C-terminus, goat and human appear to be more similar in this respect. IFs based on goat milk were also more comparable to human milk in terms of the average peptide length and in the length of the longest observed peptide. In contrast, peptides in cow milk-based IFs were significantly longer. A similar pattern was observed for β-casein derived peptides.

Considering the individual source proteins and their contribution to the overall peptidome, some major differences are evident. Goat milk-based IF contained a lower number of β-lactoglobulin, glycosylation-dependent cell adhesion molecule 1, and κ-casein derived peptides, but a higher number of lactoperoxidase-derived peptides than cow milk-based IFs. Despite the overlap of some source proteins in the peptidomes of the three species, many were species-specific and thus different. Noteworthy, no α-lactalbumin peptides were identified in any of the IF samples despite being the major whey protein in human milk. Thus, more attention should be paid to alterations induced during processing of IF. Moreover, the biological activity of endogenous peptides should be determined and compared to human milk as the observed differences in peptide profiles may differently affect the infant’s health and growth. Infants receiving IFs might be deprived from the benefits of valuable bioactive peptides naturally supplied with mother’s milk and are instead exposed to different peptides with unknown functions. Although several peptides identified here possess reportedly diverse biological activities, the current study did not aim to verify the activities of these peptides. In a next step endogenous peptides should be quantified to better evaluate the influence of processing on the peptidomes of liquid and powdered IFs. Moreover, modifications induced in endogenous peptides upon processing, e.g., Maillard reaction [13], and their effects on biological functions should be included into future studies.

## 5. Conclusions

This study compared the endogenous peptidomes of cow milk-based powdered and liquid IFs, powdered goat milk-based IF, and human milk collected from two donors in the third lactation month. The milk peptidomes of all three species represented by 1587 peptides were characterized by high numbers of β- and α_S1_-casein peptides. The other source proteins were either species-specific or were more closely related when comparing the cow and goat IF samples. Interestingly, the peptidomes of powdered and liquid cow milk-based IFs were different, i.e., α_S1_-casein peptides dominated in powdered formula while liquid IFs contained higher numbers of β-casein peptides. Most likely, this results from different processing procedures. Expectedly, the human peptidomes were very different from goat and cow milk peptidomes but appeared also to be donor-specific. The major differences observed were related to peptides derived from perilipin-3 and bile salt-activated lipase. However, further studies considering more donors are required to draw more precise conclusions.

## Data Availability

The data presented in this study are available in the article and the supplementary material. The raw data are available on request from the corresponding authors.

## Supplementary Materials

The following are available online at https://www.mdpi.com/article/10.3390/foods10112579/s1, Figure S1: Sequence coverage of β-casein in goat milk-based IF, Figure S2: Sequence coverage of β-casein in cow milk-based IF, Figure S3: Sequence coverage of β-casein in human milk, Figure S4: Sequence logo cleavage sites of β-casein, Figure S5: Distribution of the peptide length in β-casein and α_S1_-casein, Table S1: Nutritional parameters of the analyzed IF title, Table S2: Donor-specific parameters, Table S3: List of chemicals, Table S4: List of native peptides identified in the two brands of powdered goat milk based IF, Table S5: List of native peptides identified in the two brands of powdered cow milk based IF (P1 and P2) and in liquid cow milk based IF (L1 and L2), Table S6: List of native peptides identified in the two donated human milk samples.
