# Supplementary material for: Analysis of the Endogenous Peptidomes of Different Infant Formula Types and Human Milk
Source: Foods. 2021 Oct 26;10(11):2579. doi: 10.3390/foods10112579 (PMC8623676; doi:10.3390/foods10112579)
Supplement: Supplementary file 1 [file foods-10-02579-s001.zip › foods-1394980-supplementary.pdf]

Supplementary Material

Analysis of the endogenous peptidomes of different infant formula types and human milk

Michele Wölk[a,b], Corinna Gebauer[c], Ralf Hoffmann[a,b]* and Sanja Milkovska-Stamenova[a,b]*

[a] Institute of Bioanalytical Chemistry, Faculty of Chemistry and Mineralogy, Universität Leipzig, Deutscher Platz 5, 04103, Leipzig, Germany

[b] Center for Biotechnology and Biomedicine, Universität Leipzig, Deutscher Platz 5, 04103, Leipzig, Germany

[c] Department of Paediatrics, Division of Neonatology, University of Leipzig Medical Center, Liebigstr. 20 a, 04103 Leipzig, Germany

Figure S1: Sequence coverage of β-casein in goat milk-based IF……………………………..2

Figure S2: Sequence coverage of β-casein in cow milk-based IF……………………………..3

Figure S3: Sequence coverage of β-casein in human milk……….……………………………..4

Figure S4: Sequence logo cleavage sites of β-casein …………………………………….…….5

Figure S5: Distribution of the peptide length in β-casein and α_{S1}-casein………………………6

Signal Peptide

Pos	1	2	3	4	5	6	7	8	9	10	11	12	13	14	15	16	17	18	19	20	21	22	23	24	25	26	27	28	29	30
AA	M	K	V	L	I	L	A	C	L	V	A	L	A	I	A	R	E	Q	E	E	L	N	V	V	G	E	T	V	E	S
Total																10	11	11	11	11	11	11	11	10	8	7	6	5	5	4
G1																9	10	10	10	10	10	10	10	9	7	6	5	4	4	3
G2																10	11	11	11	11	11	11	11	10	8	7	6	5	5	4

Pos	31	32	33	34	35	36	37	38	39	40	41	42	43	44	45	46	47	48	49	50	51	52	53	54	55	56	57	58	59	60
AA	L	S	S	S	E	E	S	I	T	H	I	N	K	K	I	E	K	F	Q	S	E	E	Q	Q	Q	T	E	D	E	L
Total	4	4	4	5	5	5	5	5	5	5	5	5	5	7	9	9	11	13	13	13	13	13	13	13	13	13	14	13	13	13
G1	3	3	3	4	4	4	4	4	4	4	4	4	4	7	9	9	11	13	13	13	13	13	13	13	13	13	13	12	12	12
G2	4	4	4	5	5	5	5	5	5	5	5	5	5	6	8	8	10	12	12	12	12	12	12	12	12	12	13	12	12	12

Pos	61	62	63	64	65	66	67	68	69	70	71	72	73	74	75	76	77	78	79	80	81	82	83	84	85	86	87	88	89	90
AA	Q	D	K	I	H	P	F	A	Q	A	Q	S	L	V	Y	P	F	T	G	P	I	P	N	S	L	P	Q	N	I	L
Total	13	13	13	5	5	4	3	2	2	1																				
G1	12	12	12	4	4	3	3	2	2	1																				
G2	12	12	12	4	4	4	3	2	2	1																				

Pos	91	92	93	94	95	96	97	98	99	100	101	102	103	104	105	106	107	108	109	110	111	112	113	114	115	116	117	118	119	120
AA	P	L	T	Q	T	P	V	V	V	P	P	F	L	Q	P	E	I	M	G	V	P	K	V	K	E	T	M	V	P	K
Total		3	4	4	4	4	4	12	13	13	13	13	13	13	13	13	13	13	14	14	14	14	12	12	12	12	10	10	10	10
G1		3	4	4	4	4	4	11	12	12	12	12	12	12	12	12	12	12	13	13	13	13	11	11	10	10	8	8	8	8
G2		3	4	4	4	4	4	11	12	12	12	12	12	12	12	12	12	12	13	13	13	13	11	11	12	12	10	10	10	10

Pos	121	122	123	124	125	126	127	128	129	130	131	132	133	134	135	136	137	138	139	140	141	142	143	144	145	146	147	148	149	150
AA	H	K	E	M	P	F	P	K	Y	P	V	E	P	F	T	E	S	Q	S	L	T	L	T	D	V	E	K	L	H	L
Total	7	7	9	9	9	9	9	9	4	4	4	4	4	4	4	4	4	4	4	4	5	5	5	5	5	5	5	4	4	4
G1	4	4	4	4	4	4	4	4																						
G2	7	7	9	9	9	9	9	9	4	4	4	4	4	4	4	4	4	4	4	4	5	5	5	5	5	5	5	4	4	4

Pos	151	152	153	154	155	156	157	158	159	160	161	162	163	164	165	166	167	168	169	170	171	172	173	174	175	176	177	178	179	180
AA	P	L	P	L	V	Q	S	W	M	H	Q	P	P	Q	P	L	S	P	T	V	M	F	P	P	Q	S	V	L	S	L
Total	4	4	4	1	1	1	1	2	1	1	1	1	1	1	1	1	1	1	1									2	9	10
G1								1	1	1	1	1	1	1	1	1	1	1	1									2	9	10
G2	4	4	4	1	1	1	1	1																				1	3	3

Pos	181	182	183	184	185	186	187	188	189	190	191	192	193	194	195	196	197	198	199	200	201	202	203	204	205	206	207	208	209	210
AA	S	Q	P	K	V	L	P	V	P	Q	K	A	V	P	Q	R	D	M	P	I	Q	A	F	L	L	Y	Q	E	P	V
Total	12	12	12	12	29	30	30	30	30	30	29	38	38	38	39	37	37	36	36	35	35	29	27	24	24	29	31	34	37	41
G1	12	12	12	12	26	27	27	27	27	27	26	34	34	34	35	33	33	32	32	31	31	25	23	20	20	25	27	30	33	37
G2	4	4	4	4	20	20	20	20	20	20	20	28	28	28	28	28	28	27	27	26	26	22	20	21	22	26	28	31	33	37

Pos	211	212	213	214	215	216	217	218	219	220	221	222
AA	L	G	P	V	R	G	P	F	P	I	L	V
Total	43	46	48	49	49	48	48	47	47	44	31	16
G1	39	42	44	45	45	44	44	43	43	40	27	13
G2	39	42	43	44	44	43	43	43	43	41	29	16

Figure S1: Sequence coverage of β casein (86.0 % excluding the signal peptide) derived from all peptides identified in goat milk-based IF (total) and the individual brands G1 and G2.

Signal Peptide (positions 1–15)

Pos	1	2	3	4	5	6	7	8	9	10	11	12	13	14	15	16	17	18	19	20	21	22	23	24	25	26	27	28	29	30
AA	M	K	V	L	I	L	A	C	L	V	A	L	A	L	A	R	E	L	E	E	L	N	V	P	G	E	I	V	E	S
Total																4	4	4	4	4	4	4	4	4	4	4	4	4	4	4
P1																4	4	4	4	4	4	4	4	4	4	4	3	3	3	3
P2																4	4	4	4	4	4	4	4	4	4	4	3	3	3	3
L1																4	4	4	4	4	4	4	4	4	4	4	4	4	4	4
L2																4	4	4	4	4	4	4	4	4	4	4	4	4	4	4

Pos	31	32	33	34	35	36	37	38	39	40	41	42	43	44	45	46	47	48	49	50	51	52	53	54	55	56	57	58	59	60
AA	L	S	S	S	E	E	S	I	T	R	I	N	K	K	I	E	K	F	Q	S	E	E	Q	Q	Q	T	E	D	E	L
Total	5	8	9	14	15	16	16	16	16	16	16	16	13	23	28	28	29	31	31	31	31	31	31	31	30	30	30	30	29	29
P1	3	6	7	9	10	10	10	10	10	10	11	11	8	18	21	21	22	24	24	24	24	24	24	24	23	22	22	22	21	21
P2	3	3	4	6	7	7	7	7	7	7	8	8	6	16	19	19	20	22	22	22	22	22	22	22	21	19	19	19	19	19
L1	4	5	6	9	10	10	10	10	10	10	11	11	10	21	26	26	27	29	29	29	29	29	29	29	28	27	27	27	27	27
L2	5	6	7	12	13	14	14	14	14	14	14	14	12	23	28	28	29	31	31	31	31	31	31	31	30	29	29	29	28	28

Pos	61	62	63	64	65	66	67	68	69	70	71	72	73	74	75	76	77	78	79	80	81	82	83	84	85	86	87	88	89	90
AA	Q	D	K	I	H	P	F	A	Q	T	Q	S	L	V	Y	P	F	P	G	P	I	P	N	S	L	P	Q	N	I	P
Total	29	30	30	24	24	23	22	18	13	12	10	6																1	1	1
P1	21	22	22	16	16	15	15	11	9	8	7	3																1	1	1
P2	19	20	20	14	14	13	12	9	8	8	7	3																1	1	1
L1	27	27	27	21	21	20	19	17	12	11	9	6																		
L2	28	28	28	22	22	21	20	17	12	11	9	6																1	1	1

Pos	91	92	93	94	95	96	97	98	99	100	101	102	103	104	105	106	107	108	109	110	111	112	113	114	115	116	117	118	119	120
AA	P	L	T	Q	T	P	V	V	V	P	P	F	L	Q	P	E	V	M	G	V	S	K	V	K	E	A	M	A	P	K
Total	1	1	1	1	1	1	1	1	3	3	3	3	3	3	3	4	5	7	8	8	8	8	9	9	9	9	11	11	11	10
P1	1	1	1	1	1	1	1	1	3	3	3	3	3	3	3	3	4	4	4	4	3	3	4	4	4	4	4	4	4	4
P2	1	1	1	1	1	1	1	1	3	3	3	3	3	3	3	3	4	4	5	5	3	3	4	4	4	4	4	4	4	3
L1									2	2	2	2	2	2	2	2	3	4	5	5	5	5	5	5	5	5	7	7	7	7
L2	1	1	1	1	1	1	1	1	2	2	2	2	2	2	2	3	4	6	7	7	7	7	8	8	8	8	8	8	8	7

Pos	121	122	123	124	125	126	127	128	129	130	131	132	133	134	135	136	137	138	139	140	141	142	143	144	145	146	147	148	149	150
AA	H	K	E	M	P	F	P	K	Y	P	V	E	P	F	T	E	S	Q	S	L	T	L	T	D	V	E	N	L	H	L
Total	14	14	16	18	18	18	18	18	17	17	17	17	17	17	14	14	14	14	11	8	3	4	4	4	4	4	5	5	6	7
P1	10	10	11	13	13	13	13	13	12	13	13	13	13	13	10	10	10	10	9	6	2	2	2	2	2	2	3	3	3	4
P2	8	8	9	11	11	11	11	11	10	10	10	10	10	10	7	7	7	7	7	4	1	1	1	1	1	1	2	2	3	4
L1	10	10	11	11	11	11	11	11	11	13	13	13	13	13	12	12	12	12	9	6	2	3	3	3	3	3	3	3	3	3
L2	7	7	8	8	8	8	8	8	7	8	8	8	8	8	8	8	8	8	7	5	3	4	4	4	4	4	4	4	4	4

Pos	151	152	153	154	155	156	157	158	159	160	161	162	163	164	165	166	167	168	169	170	171	172	173	174	175	176	177	178	179	180
AA	P	L	P	L	L	Q	S	W	M	H	Q	P	H	Q	P	L	P	P	T	V	M	F	P	P	Q	S	V	L	S	L
Total	7	7	7	15	17	17	19	21	21	21	20	18	17	16	15	14	14	14	13	5	4	3	4	4	4	6	21	26	39	47
P1	4	4	4	3	3	3	3	3	2	2	2	2	2	2	2	2	2	2	2	1	1	1	1	1	1	2	3	6	12	14
P2	4	4	4	4	4	4	4	4	4	4	4	4	4	4	4	4	4	4	4	2	2	1	2	2	2	3	3	8	18	21
L1	3	3	3	10	12	12	14	16	18	18	18	16	15	14	13	12	12	12	11	3	2	2	2	2	2	4	18	21	32	39
L2	4	4	4	9	9	9	9	9	10	10	9	7	6	5	4	4	4	4	4							1	14	18	27	36

Pos	181	182	183	184	185	186	187	188	189	190	191	192	193	194	195	196	197	198	199	200	201	202	203	204	205	206	207	208	209	210
AA	S	Q	S	K	V	L	P	V	P	Q	K	A	V	P	Y	P	Q	R	D	M	P	I	Q	A	F	L	L	Y	Q	E
Total	61	62	66	68	69	70	70	68	68	66	68	71	77	78	82	82	80	71	71	71	71	62	62	54	37	22	12	12	13	17
P1	18	19	20	21	23	25	25	25	25	25	26	26	30	30	31	31	31	22	22	22	22	21	21	20	14	4	5	7	8	12
P2	28	29	32	33	35	36	36	36	36	35	38	39	42	42	43	43	43	35	35	35	35	33	33	31	21	10	10	10	11	15
L1	51	52	55	57	59	59	59	57	57	55	57	59	65	66	70	70	68	59	59	59	59	50	50	42	25	19	9	12	13	15
L2	43	43	45	46	48	48	48	48	48	46	50	50	51	51	51	51	49	42	42	42	42	38	38	36	27	14	10	11	11	15

Pos	211	212	213	214	215	216	217	218	219	220	221	222	223	224
AA	P	V	L	G	P	V	R	G	P	F	P	I	I	V
Total	19	21	22	25	26	26	26	25	25	25	25	19	15	12
P1	12	12	12	14	15	15	15	14	14	14	14	11	10	8
P2	16	17	18	21	22	22	22	21	21	21	21	17	14	11
L1	17	19	20	22	23	23	23	23	23	23	23	17	14	12
L2	17	17	17	19	20	20	20	19	19	19	19	14	11	9

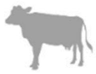

Figure S2: Sequence coverage of β casein (92.5 % excluding the signal peptide) derived from all peptides identified in all samples of cow milk-based IFs (total) and in each brand of powdered (P1 and P2) and liquid cow milk-based IF (L1 and L2).

								Signal Peptide																						
Pos	1	2	3	4	5	6	7	8	9	10	11	12	13	14	15	16	17	18	19	20	21	22	23	24	25	26	27	28	29	30
AA	M	K	V	L	I	L	A	C	L	V	A	L	A	L	A	R	E	T	I	E	S	L	S	S	S	E	E	S	I	T
Total																29	39	41	43	45	51	52	59	60	63	65	66	66	66	66
D1																25	33	34	35	36	42	43	47	48	50	52	53	53	53	53
D2																28	38	40	42	44	50	51	58	59	62	64	65	65	65	65

Pos	31	32	33	34	35	36	37	38	39	40	41	42	43	44	45	46	47	48	49	50	51	52	53	54	55	56	57	58	59	60
AA	E	Y	K	Q	K	V	E	K	V	K	H	E	D	Q	Q	Q	G	E	D	E	H	Q	D	K	I	Y	P	S	F	Q
Total	66	65	65	49	50	44	42	41	27	27	25	24	25	25	25	25	23	22	22	22	22	22	22	20	15	15	12	6	3	2
D1	53	52	52	38	39	34	33	33	22	22	20	19	20	20	20	20	18	17	17	17	17	17	17	17	12	12	10	4	1	1
D2	65	64	64	48	49	43	41	40	26	26	24	24	25	25	25	25	23	22	22	22	22	22	22	20	15	15	12	6	3	2

Pos	61	62	63	64	65	66	67	68	69	70	71	72	73	74	75	76	77	78	79	80	81	82	83	84	85	86	87	88	89	90
AA	P	Q	P	L	I	Y	P	F	V	E	P	I	P	Y	G	F	L	P	Q	N	I	L	P	L	A	Q	P	A	V	V
Total	2	2	2	1	1	1	1	1	1	1	1	1	1	1	1	1	1	1	1	1	2	2	4	5	7	9	9	11	14	15
D1	1	1	1												1	1	1	1	1	1	1	1	1	1	1	1	1	2	3	3
D2	2	2	2	1	1	1	1	1	1	1	1	1	1	1	1	1	1	1	1	1	2	2	4	5	7	9	9	11	14	15

Pos	91	92	93	94	95	96	97	98	99	100	101	102	103	104	105	106	107	108	109	110	111	112	113	114	115	116	117	118	119	120
AA	L	P	V	P	Q	P	E	I	M	E	V	P	K	A	K	D	T	V	Y	T	K	G	R	V	M	P	V	L	K	S
Total	16	17	17	17	17	17	18	18	18	19	20	20	20	23	26	32	32	31	32	32	27	21	22	24	29	29	29	29	24	17
D1	3	3	3	3	3	3	3	3	3	4	5	5	5	9	11	13	13	12	12	12	10	11	11	12	15	15	15	15	13	7
D2	16	17	17	17	17	17	18	18	18	19	20	20	20	23	26	32	32	31	32	32	27	21	22	24	29	29	29	29	24	17

Pos	121	122	123	124	125	126	127	128	129	130	131	132	133	134	135	136	137	138	139	140	141	142	143	144	145	146	147	148	149	150
AA	P	T	I	P	F	F	D	P	Q	I	P	K	L	T	D	L	E	N	L	H	L	P	L	P	L	L	Q	P	L	M
Total	17	17	17	18	17	17	18	16	16	16	16	11	1	1	1	1	1	1	1	1	1	1	1	1						
D1	7	7	7	8	8	8	9	9	9	9	9	7	1	1	1	1	1	1	1	1	1	1	1	1						
D2	17	17	17	18	17	17	18	16	16	16	16	11	1	1	1	1	1	1	1	1	1	1	1	1						

Pos	151	152	153	154	155	156	157	158	159	160	161	162	163	164	165	166	167	168	169	170	171	172	173	174	175	176	177	178	179	180
AA	Q	Q	V	P	Q	P	I	P	Q	T	L	A	L	P	P	Q	P	L	W	S	V	P	Q	P	K	V	L	P	I	P
Total																										3	4	4	4	4
D1																										2	3	3	3	3
D2																										3	4	4	4	4

Pos	181	182	183	184	185	186	187	188	189	190	191	192	193	194	195	196	197	198	199	200	201	202	203	204	205	206	207	208	209	210
AA	Q	Q	V	V	P	Y	P	Q	R	A	V	P	V	Q	A	L	L	L	N	Q	E	L	L	L	N	P	T	H	Q	I
Total	4	4	4	4	4	4	4	4	4	4	5	5	5	5	5	4	4	5	5	6	6	7	7	7	8	9	10	10	10	10
D1	3	3	3	3	3	3	3	3	3	2	3	3	3	3	4	3	3	4	4	5	5	6	6	6	7	8	9	9	9	9
D2	4	4	4	4	4	4	4	4	4	4	5	5	5	5	5	4	4	5	5	6	6	7	7	7	8	9	10	10	10	10

Pos	211	212	213	214	215	216	217	218	219	220	221	222	223	224	225	226
AA	Y	P	V	T	Q	P	L	A	P	V	H	N	P	I	S	V
Total	11	11	11	11	12	13	13	14	14	14	14	13	13	13	13	13
D1	10	10	10	10	11	12	12	13	13	13	13	13	13	13	13	13
D2	11	11	11	11	12	13	13	14	14	14	14	13	13	13	13	13

Figure S3: Sequence coverage of β-casein (85.2 % excluding the signal peptide) derived from all peptides identified in human milk (total) and for each individual sample (D1 and D2).

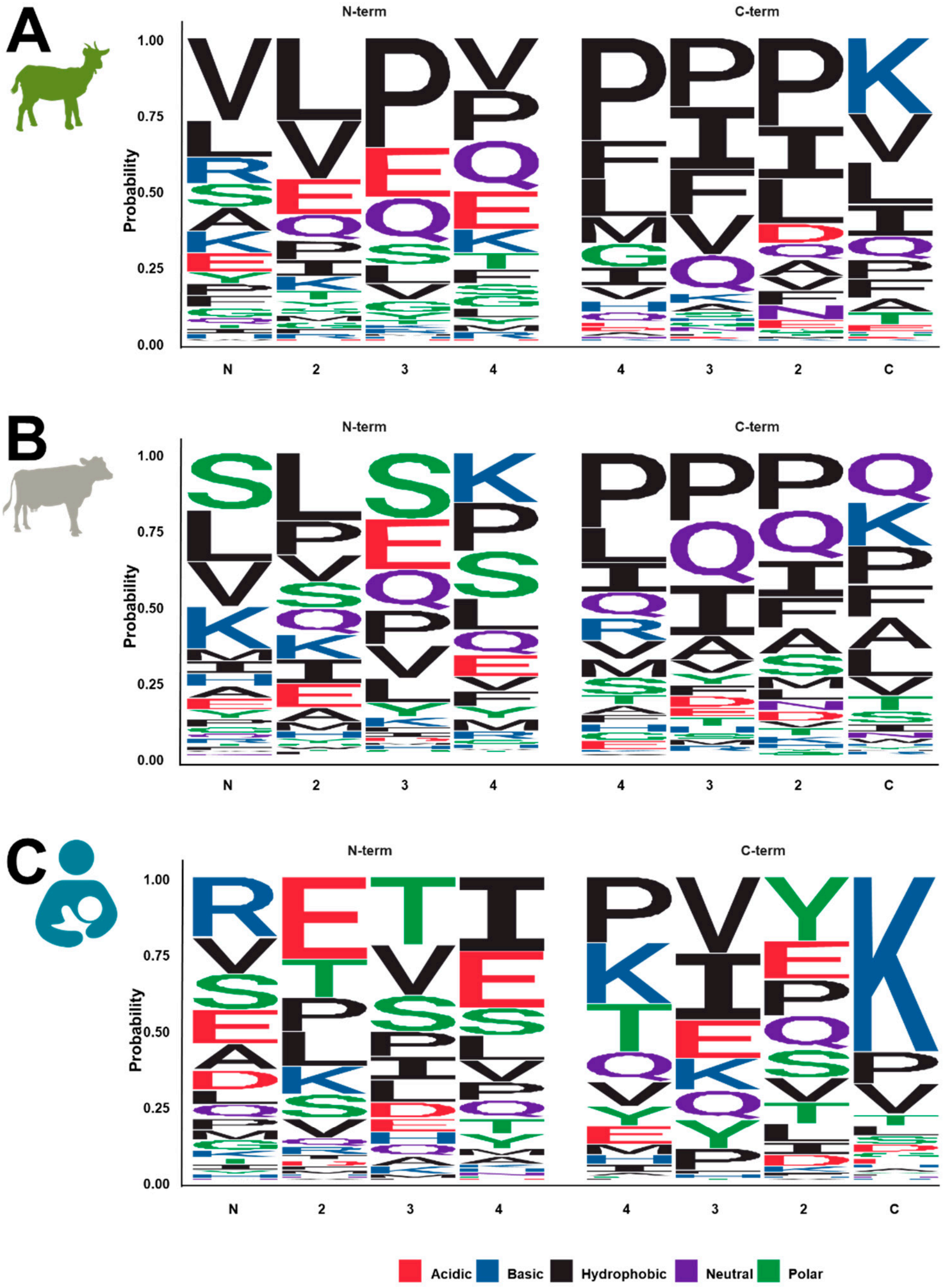

Figure S4: Sequence logo displaying cleavage patterns considering all β-casein-derived peptides identified in goat milk-based IF (A), cow milk-based IF (B), and human milk (C).

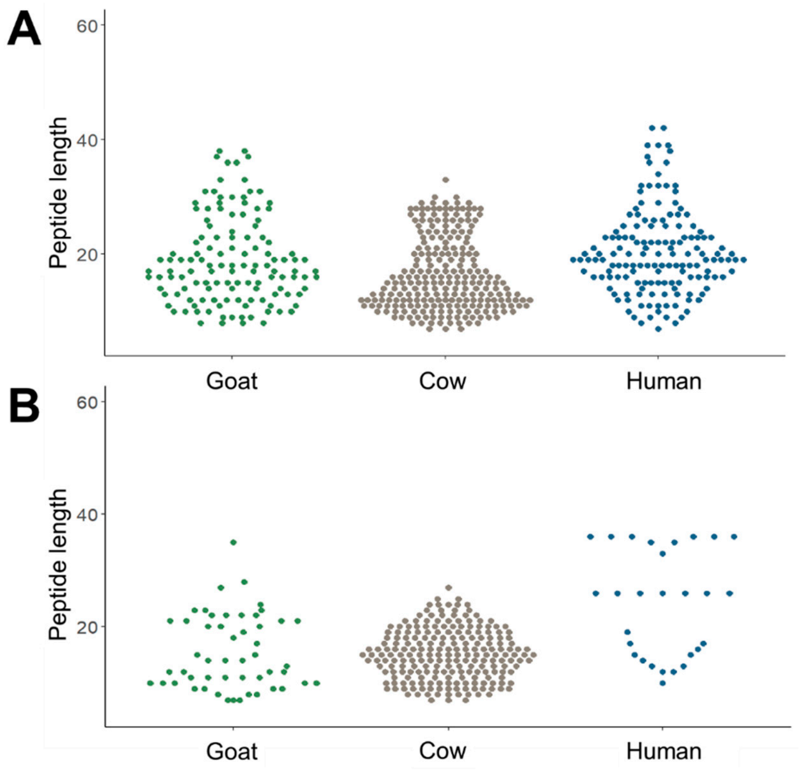

Figure S5: Distribution of the number of residues in β- (A) and α_{S1}-casein-derived peptides (B) identified in goat milk-based IF, cow milk-based IF, and human milk.
